# PINK1 Immunoexpression Predicts Survival in Patients Undergoing Hepatic Resection for Colorectal Liver Metastases

**DOI:** 10.3390/ijms24076506

**Published:** 2023-03-30

**Authors:** Juan Carlos Celis-Pinto, Adela Alonso Fernández-Velasco, María Daniela Corte-Torres, Jorge Santos-Juanes, Noelia Blanco-Agudín, Kelvin Manuel Piña Batista, Jesús Merayo-Lloves, Luis M. Quirós, Iván Fernández-Vega

**Affiliations:** 1 Department of Pathology, Central University Hospital of Asturias (HUCA), University of Oviedo, 33011 Oviedo, Spain; juancarlos.celis@outlook.com (J.C.C.-P.); ade21_1991@hotmail.com (A.A.F.-V.); 2 Biobank of Principality of Asturias, 33011 Oviedo, Spain; 3 Health Research Institute of the Principality of Asturias (ISPA), 33011 Oviedo, Spain; 4 Department of Dermatology, Central University Hospital of Asturias (HUCA), 33011 Oviedo, Spain; 5 Department of Functional Biology, University of Oviedo, 33006 Oviedo, Spain; 6 Department of Neurosurgery, Central University Hospital of Asturias (HUCA), 33011 Oviedo, Spain; 7 Instituto Universitario Fernández-Vega, 33012 Oviedo, Spain

**Keywords:** PINK1, adenocarcinoma, immunohistochemistry, metastasis, surgery

## Abstract

PTEN-induced kinase-1 (PINK1) is the initiator of the canonical mitophagy pathway. Our aim was to study the immunoexpression of PINK1 in surgical specimens from ninety patients with metastatic colorectal adenocarcinoma (CRC) to the liver (CRLM). Tissue arrays were produced, and immunohistochemical studies were analyzed by the H-Score method. The mean immunoexpression of PINK1 in normal tissues was between 40 to 100 points. In tumoral tissues, positive PINK1 immunoexpression was observed in all samples, and no differences were noted between CRCs. In CRLMs, a significant under-expression was noted for PINK1 from the rectum (71.3 ± 30.8; *p* < 0.042) compared to other sites. Altered PINK1 immunoexpression in CRCs, either higher than 100 points or lower than 40 points, was associated with worse overall survival (OS) (*p* < 0.012) due to a shorter post-metastatic survival (PMS) (*p* < 0.023), and it was found to be a significant independent predictor of prognosis in a multivariate model for OS and PMS (HR = 1.972, 95% CI 0.971–4.005; *p* = 0.022. HR = 2.023, 95% CI 1.003–4.091; *p* = 0.037, respectively). In conclusion, altered PINK1 immunoexpression determined in CRCs with resected CRLM predicts a worse prognosis, possibly due to the abnormal function of mitophagy.

## 1. Introduction

Colorectal cancer (CRC) is the third most common type of cancer in the world and ranks second in terms of mortality [1,2]. Most CRCs are adenocarcinomas that may spread and invade different distant organs through the blood and lymphatic circulation and give rise to metastatic lesions, especially to the liver (CRLM) [3,4,5]. Approximately half of all CRCs will relapse with liver metastases during follow-up, which equates to a poor prognosis [6]. Surgical resection of CRLM is a potentially curative option, and advances in surgical techniques and chemotherapy have improved the prognosis of patients with CRLM and may lead to better outcomes in CRC patients [7]. Unfortunately, only about 25% of CRLM are resectable at initial presentation [8]. However, there is a subset of patients with CRLM that can benefit from radical surgery; almost 50% of them can survive five years after surgery or possibly even achieve a cure [9,10]. Discovering biological differences in CRC is essential to explain the variability in prognosis.

Mitophagy plays an important homeostatic function in cells and tissues, maintaining the integrity of the mitochondrial pool by eliminating old and/or damaged mitochondria [11,12]. Therefore, defects in mitophagy could lead to a failure in proper reprogramming of cellular metabolism, control of cell fate determination, attenuation of inflammation and response to DNA damage [12]. PTEN-induced kinase-1 (PINK1) is the initiator of the canonical mitophagy pathway, the PINK1/Parkin-mediated mitophagy. PINK1 is expressed ubiquitously and localizes to cytosol and mitochondria [13]. Recently, increasing attention has been drawn toward PINK1 in several diseases, including cancer [14,15,16,17]. Thus, the PINK1 signaling system has been shown to control several processes key to cancer cell biology, especially in mitochondrial homeostasis and dynamics, including bioenergetics, mitophagy, fission and fusion. However, much current evidence about PINK1 addresses a duality of its function and may stem from its role in regulating mitophagy, which may be tumor-promoting in some circumstances and tumor suppressive in others, depending on the cellular context [14]. Accumulating reports suggest that dysregulation of mitophagy contributes to neoplastic progression and drug resistance in various types of tumors [18]. Recent studies in gastric cancer showed that PINK1 deficiency compromises mitophagy, promotes the Warburg effect, and facilitates the M2 polarisation of macrophages [19]. Besides, PINK1 may contribute to chemoresistance in non-small cell lung cancer [20]. Eventually, PINK1 has the potential as a biomarker for prognosis and the immune response across cancers [21].

In the present study, we aimed to investigate the possible clinical relevance of PINK1, one of the major regulator proteins of mitophagy, in suitable samples from patients with CRC and resected CRLM.

## 2. Results

### 2.1. Clinicopathological Features of Cases

Ninety patients of CRC with curative resection (R0 resection) of CRLM and suitable tumor material were enrolled in the study with a follow-up of fifteen years. Seventy-eight patients had metachronous liver metastases (86.7%). No consideration was taken if the patient had single or multiple uni and/or bilobar liver metastases. Concerning postoperative morbidity of liver resections, only one patient died during the first month after surgery (1, 1%). Adjuvant or neoadjuvant therapy was developed following up-to-date guidelines according to state of art therapies.

Patients were divided into three groups depending on primary localization and main clinicopathological features, as summarized in Table 1. Except for two factors, such as gender and rectum adenocarcinomas, there was no significant difference between the three groups with regard to patient characteristics. Rectum adenocarcinomas were significantly smaller than colon adenocarcinomas (*p* = 0.029), mostly treated with neoadjuvant therapy (*p* < 0.001) and showed more necrotic tissue than other localizations (*p* = 0.004). Non-significant differences between groups were noted concerning age, status, nodal stage, tumor stage, survival, grade of differentiation and mitotic activity. The pathological extramural venous invasion was not determined in our sample because all patients already have metastasis. Tumors of the RAS mutant-type group were observed in the proximal colon significantly more frequently than in the distal colon or rectum (*p* = 0.045). *RAS* somatic mutations were detected in 31 cases out of 56 examined (28 *KRAS* and 3 *NRAS*). BRAF cases were not found.

A comparative study of CRCs and their liver metastasis pointed out that those from the right colon were mostly similar for TB, grade of differentiation, necrosis, and mitotic activity. On the other hand, liver metastasis from the left-sided colon showed the highest differences with the primary tumoral tissue for TB and tumoral grade (Table 2).

A significant strong positive correlation between OS and PMS was noted (r = 0.863; *p* < 0.001). A significant moderate correlation between OS and DFS was also noted (r = 0.412; *p* < 0.001). Furthermore, the patient’s age showed a significant weak positive correlation with mitotic activity in mCRC (r = 0.227; *p* = 0.035) and a negative correlation with DFS (r = −0.213; *p* = 0.044) (Table 3).

Additionally, Kaplan-Meier survival analysis pointed out that the group of high budding in CRC showed significantly less OS (mean OS: 70.7 ± 8.0 months versus 132.2 ± 22.5 months; *p* = 0.011), DFS (mean OS: 30.4 ± 5.6 months versus 66.8 ± 13.9 months; *p* = 0.012) and PMS (mean OS: 56.7 ± 7.9 months versus 106.3 ± 20.7 months; *p* = 0.016). No significant differences were noted when the analysis of TB was performed in the CRLM. In the current study, patients with all-*RAS* mutations did not show significantly worse survival rates in comparison to all-*RAS* wild-type patients.

### 2.2. Immunohistochemical Study of PINK1

**Figure 1:**
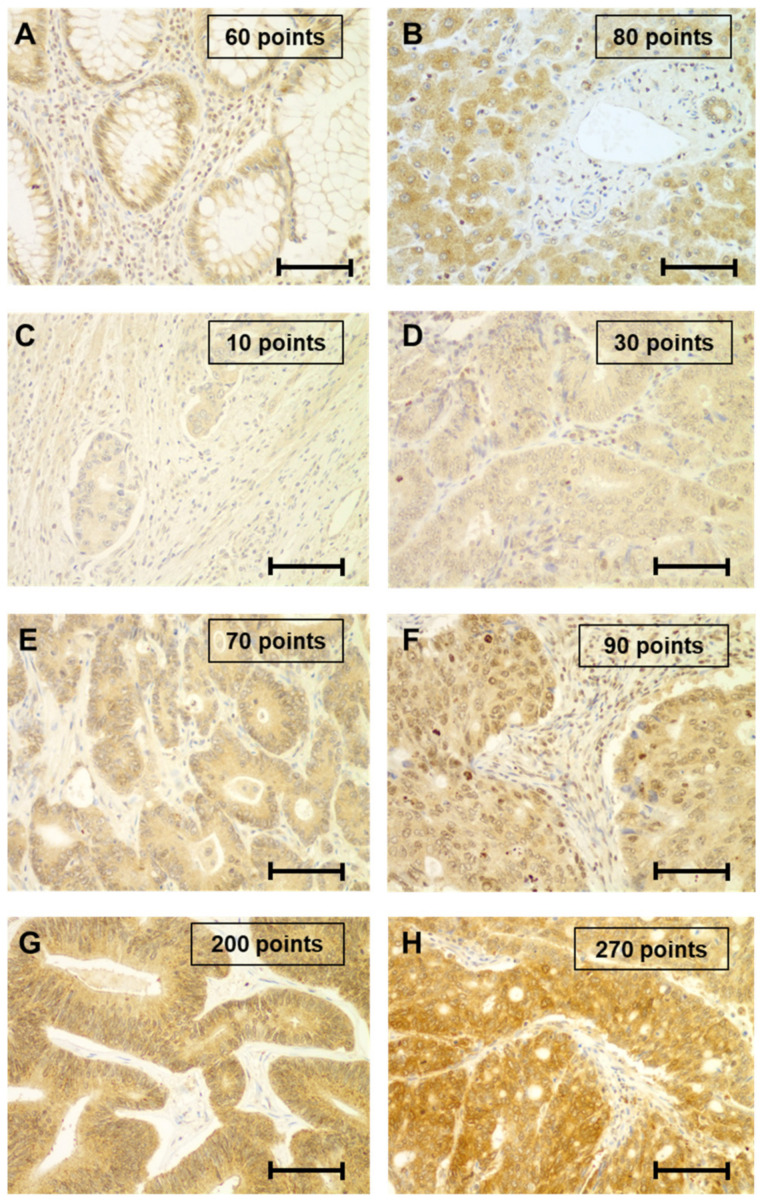
Immunostaining for PINK1 protein in normal (colon/ liver) and tumoral (CRC/ CRLM) tissues. Images of normal tissues of the colon (**A**) and liver (**B**) showing H-score values between 40 to 100 points. Pictures of typical cases of CRC and CRLM showing H-score values lower than 40 points (**C**,**D**), between 40 to 100 points (**E**,**F**) and higher than 100 points (**G**,**H**), respectively. Scale bars in (**A**–**H**) = 40 μm.

A mean immunoexpression of 76.34 ± 13.7 points for colon mucosa cells and 92.56 ± 17.2 points for the hepatocytes was determined, all results being between 40 to 100 points. Variable cytoplasmic staining was noted in both absorptive and goblet cells from the colonic mucosa and hepatocytes and biliary epithelial cells in the liver. Some inflammatory cells showed positivity for PINK1. Fibroblasts were predominantly negative (Figure 1A,B).

In tumor tissues, all samples were positive for PINK1 but with high variability. Analysis and characterization of PINK1 immunohistochemical expression in the surrounding tumoral microenvironment was not intended in this work. In CRCs, PINK1 showed a mean expression of 82.3 ± 48.7 points with no significant differences either with normal colon mucosa or between groups of adenocarcinomas based on primary localization. On the contrary, significant differences were noted for PINK1 in CRLM depending on primary origin (*p* = 0.018) (Table 1). Concerning PINK1 immunoexpression in CRC and CRLM by localization, significant overexpression was noted in CRLM samples from the right-sided colon (mean of 105.0 ± 70.8 points versus 79.0 ± 49.9 points, respectively; *p* = 0.012) and left-sided colon (mean of 95.5 ± 42.1 points versus 76.4 ± 39.6 points, respectively; *p* = 0.015) (Table 2). Moreover, a significant moderate positive correlation between PINK1 immunoexpression in CRC and CRLM was observed (r = 0.352; *p* < 0.001) (Table 3) (Figure 1C–H).

### 2.3. Survival Curves, Univariate and Multivariate Analysis Concerning PINK1

**Figure 2:**
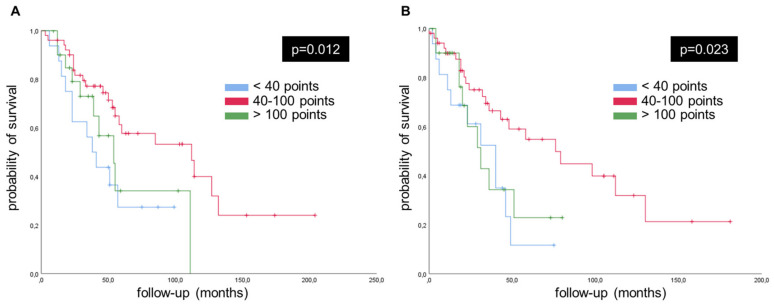
Kaplan-Meier estimates survivals for PINK1 in CRC with resected CRLM. Those PINK1 cases with H-score values between 40 to 100 points showed better OS (**A**) and PMS (**B**).

Kaplan-Meier survival analysis revealed a significantly better OS (*p* < 0.012) and PMS (*p* < 0.023) for the 58% of patients with PINK1 immunoexpression in CRCs tested, which were in ranges comparable to the colon mucosa (mean OS: 103.6 ± 12.9 months; mean PMS: 86.1 ± 11.9 months). On the other hand, samples with values either under 40 points or above 100 points of PINK1 highlighted a significantly worse OS (Score < 40 points = mean OS: 49.2 ± 8.6 months; Score > 100 points = mean OS: 62.0 ± 11.0 months) and PMS (Score < 40 points = mean OS: 33.4 ± 5.9 months; Score > 100 points = mean OS: 38.7 ± 7.1 months) (Figure 2). Survival analysis of PINK1 immunoexpression performed on CRLM samples did not show significant results.

Univariate analysis showed that the presence of neoadjuvant therapy (*p* = 0.041), poor grade of differentiation in CRC (*p* = 0.024) and altered PINK1 expression in CRC (HR = 2.09, 95% CI 1.035–4.219; *p* = 0.015) were associated with poor OS. Moreover, altered PINK1 expression in CRC (HR = 2.055, 95% CI 1.014–4.164; *p* = 0.026) was associated with poor PMS. On the other hand, low TB was correlated with better OS and PMS (*p* = 0.026 and *p* = 0.022, respectively). Furthermore, we evaluated the prognostic information obtained by the addition of neoadjuvant therapy and grade in CRC and TBs to a multivariable model in terms of OS and PMS. Therefore, altered PINK1 expression was found to be a significant independent predictor in a multivariate model for OS and PMS (HR = 1.972, 95% CI 0.971–4.005; *p* = 0.022. HR = 2.023, 95% CI 1.003–4.091; *p* = 0.037, respectively) (Table 4).

## 3. Discussion

In the past few decades, resection of metastatic tissue with curative intent has been more often considered as a multimodality approach together with systemic treatment, especially in CRLM [7,22]. Variations in metastatic tissue with clinical implications either at the histological and/or molecular level have been previously described [23,24,25]. In this work, we have studied the immunohistochemical expression of the kinase PINK1 in a specific subset of patients with colorectal adenocarcinomas, consisting only of those patients who underwent curative surgical resection of their CRLM, to better describe molecular alterations in CRCs at this stage of advanced disease.

Histopathological examination showed that samples from the rectum were significantly smaller and showed higher levels of necrosis, possibly due to neoadjuvant therapy [26]. Survival analysis indicated that tumor budding assessment in CRC was significantly associated with survival, as previously described in the literature [27]. In relation to CRLM cases, TB analysis did not show significant results in terms of survival. Fonseca et al. described that TB in CRLM is a prognostic factor, but it is not an independent predictor of survival [28]. Blank et al. implemented the terms intra-metastatic TB (IMB) and peri-metastatic TB (PMB) and mostly concluded that TB assessment in CRLM is challenging in specific cases; therefore, they suggested evaluating TB only in metastases with desmoplastic stroma reaction [29]. In the present study, IMB was not considered in the assessment of TB in CRLM.

Approximately half of CRC patients had *RAS* mutation, and it was more frequent in the proximal colon compared with the distal colon and rectum [30,31]. *KRAS* mutations were recognized as a predictive marker of the prognosis of CRLM patients undergoing hepatic resection [30,31,32,33]. In the current study, cases with all-*RAS* mutations did not show significantly worse survival rates in comparison to all-*RAS* wild-type patients. This result could be biased by the fact that up to 48% of cases in our sample did not have molecular analysis for all-*RAS* status.

Currently, the study of potential biomarkers concerning early diagnosis, prognosis, and treatment of colorectal adenocarcinoma has increased [34]. Concerning PINK1 immunoexpression, no significant differences were noted between mCRC from a different location. For CRLMs, PINK1 under-expression was observed in samples from the rectum. This finding may be explained by a possible clonal evolution after neoadjuvant therapy, as rectum cancers also undergo radiotherapy [35,36]. Conversely, significantly higher levels of PINK1 immunoexpression were observed in CRLMs from the colon, which may be explained by a similar justification of clonal evolution after adjuvant chemotherapy that patients at risk of disease progression usually undergo [35,36]. To the best of our knowledge, this is the first study testing PINK1 in metastatic tissues by immunohistochemistry. A recent pan-cancer analysis indicated that *PINK1* mRNA expression was lower in several cancer groups than in normal tissues, including the brain, breast, colorectal, oesophageal, head and neck, liver and ovarian cancers, as well as leukemia and melanoma, and higher in diffuse large B-cell lymphoma [21]. Bednarczyk et al. observed that *PINK1* mRNA expression was variable depending on the four clinical stages, being reduced only in stage II, while in other stages, it showed overexpression [37].

Hajar et al. described a significant down-regulation of *PINK1* mRNA in breast cancer samples compared with nearby tissues in association with mitotic rate [38]. In non-small-cell lung cancer (NSCLC), *PINK1* was significantly overexpressed in NSCLC tissues and NSCLC cell lines and correlated with clinical pathologic variables of NSCLC [20].

Survival analysis pointed out that preserved PINK1 levels in mCRC with resected CRLM were significantly correlated with better OS because of a much better PMS. In this light, altered levels of PINK1 pointed out two groups of patients with significantly worse survival, such as those with H-Scores less than 40 points or more than 100 points. In addition, we observed that altered PINK expression was found to be a significant independent predictor in a multivariate model for OS and PMS. In advanced cancer disease, the molecular profile is increasing in relevance and sometimes weighs more than clinical and/or histopathological features [39,40,41]. Although the role of PINK1 across cancers remains unclear, previous studies indicated that PINK1 influenced the prognosis of patients in some specific cancers. Thus, PINK1 played a protective role in blood cancer, brain cancer, breast cancer, lung cancer, and soft tissue cancer and a detrimental role in colorectal cancer [21]. Kulun et al. demonstrated that PINK1 suppresses colon tumor growth by metabolic reprogramming. Furthermore, they also highlighted that PINK1 disruption simultaneously increased xenografted tumor growth [42]. In this sense, it has been described that PINK1 deficiency reprogramed glucose metabolism through HIF1α to maintain cell proliferation and even cancer growth, especially in glioblastoma [43,44]. On the other hand, silencing of PINK1 repressed cell growth and migration and induced apoptosis of lung cancer cells [15]. Then, in lung cancer cases, Xiao et al. recently described that PINK1 overexpression promoted cell migration and proliferation and predicted a poor prognosis [45]. Taking it all together, PINK1 alteration might be associated with carcinogenesis due to mitophagy dysfunction but depending on some specific tumors. To go further beyond, Metformin (Met) therapy can stimulate mitophagy by activating mitochondrial PINK1/Parkin signaling. This effect would restore PINK1 levels, especially in those mCRC cases with low expression, apart from other well-known antitumor properties [46,47].

We understand there are several limitations in our work. First, there are potential biases due to their retrospective nature. Second, the study is limited to a specific subset of CRC and the determination of PINK1 immunoexpression in non-metastatic colorectal adenocarcinomas was not addressed. Third, these findings are based on patients treated in a University Hospital; hence the percentage of poor prognostic tumors might be higher due to referral than in the daily ambulatory practice. Fourth, the study was performed in a unique center. Fifth, immunohistochemical analysis was carried out using tissue microarrays; however, the PINK1 immunoexpression pattern was quite homogeneous and concordant in the three representative tissue cores selected from each tumor.

## 4. Materials and Method

### 4.1. Patients and Samples

A total of 90 patients with suitable biopsies of CRC adenocarcinomas and resected CRLM treated between 2005 and 2020 with curative intent were retrospectively selected from the Department of Pathology electronic database at the Hospital Universitario Central de Asturias. All the electronic medical records were reviewed to determine whether outcomes of interest occurred. All the tumors were excised with conventional surgery. Patients with positive margins or limited samples were excluded. The original archived H and E slides were reviewed by a pathologist, and diagnoses were established following the latest WHO guideline [48]. Information about the tumor stage was obtained from the date of the diagnosis of CRC. Clinical patient-related data were collected. Patient age was defined as the age at the time of colorectal resection. Information concerning microsatellite instability and all-*RAS* mutation status was obtained when available [49]. Ethics approval was obtained from the Hospital Universitario Central de Asturias Committee, and the study was performed in accordance with the Declaration of Helsinki.

### 4.2. Histopathologic Evaluation

Each sample was analyzed by two independent observers (and a third one in the case of strong disagreement, which only happened on two occasions.) and registered the following histopathologic features using hematoxylin-eosin-stained slides: degree of differentiation classified as well differentiated (1), moderately differentiated (2) and poorly differentiated (3); absence or presence and percentage of necrosis, mitotic activity by 10 high power fields and tumor budding (TB). Tumor budding is defined as the presence of single tumor cells or small clusters of up to 5 cells in the tumor stroma ahead of the invasive tumor front. The intensity of TB was classified as none/low (<10 buds) or high (≥10 buds) in a 20× objective field, as it is shown elsewhere [49,50].

### 4.3. Tissue Microarray Construction

Tissue microarrays (TMAs) were constructed from tissue blocks used for routine pathological evaluation. Morphologically representative areas were selected from each individual tumor paraffin block. Areas in each case with the most representative histology to overcome tumor heterogeneity were selected, and three 3 mm tissue cores were taken from each donor block and extruded into the recipient array. Thus, TMAs were created containing three tissue cores from each of the 90 CRCs and their respective 90 CRLM. In addition, each TMA included two cores of normal colon and liver tissue as internal controls. A section from each microarray was stained with H&E to check the adequacy of tissue sampling. After 5 min at 60 °C, the TMA blocks were subsequently cut using a microtome into 3 μm thick sections and mounted on glass slides in preparation for immunohistochemistry.

### 4.4. Immunohistochemistry

For expression analysis by immunohistochemistry, we used the EnVision FLEX High pH (Link) Kit (Agilent-Dako, K800021, Santa Clara, CA, USA) and Dako Autostainer system. Paraffin-embedded tissue sections (3 µm) were deparaffinized, rehydrated, and epitope retrieved by heat induction (HIER) at 95 °C for 20 min and ph 9 (Agilent-Dako) in the Pre-Treatment Module, PT-LINK (Agilent-Dako). Endogenous peroxidase activity was blocked with EnVision™ FLEX Peroxidase-Blocking Reagent (DM821) for 5 min. The sections were incubated with rabbit Anti-PINK-1 polyclonal antibody (BC100-494, NobusBiologicals. Madrid. Spain) at 1:200 dilution for 30 min. The antigen-antibody reaction was detected with the Dako EnVision + Dual Link System-HRP (Agilent-Dako). The signal was detected using diaminobenzidine chromogen as substrate in Dako EnVision™ FLEX /HRP (Agilent-Dako). Counterstaining with hematoxylin was the final step. Negative controls were processed by omitting the primary antibody. Hepatic tissue was used as a positive control [51]. After the whole process, sections were dehydrated and mounted with a permanent medium (Agilent-Dako mounting medium, CS703). The sections were studied and photographed under a light microscope (Nikon, New York, NY, USA—Eclipse 80i).

### 4.5. Immunohistochemistry Assessment

Immunoexpression of PINK1 was evaluated by two independent observers (and a third one in the case of strong disagreement, which only happened on four occasions) without any prior knowledge of each patient’s clinical information and outcome. We used a semiquantitative approach called H-score (or “histo” score) as described elsewhere [52,53]. The final score gives more relative weight to higher intensity staining in each tumor sample. Then the sample can then be categorized into a qualitative variable and considered positive or negative based on a specific discriminatory threshold. Besides, the final score was the mean of the three cores analyzed for each case. In addition, excellent agreement was obtained for the immunohistochemistry assessment by the observers (k = 0.802) based on a hierarchical kappa test. Strong disagreement was considered for those cases evaluated with more than 30 points of difference. Discrepant cases were reevaluated, and the disagreement was resolved.

### 4.6. Statistical Analysis

Baseline demographic and clinical characteristics of the patients and pathological data were summarized with standard descriptive statistics. The primary endpoints were time to liver metastasis and time to death, defined as the time from the date of surgical excision of the primary tumor to the date of diagnosis of metastasis (disease-free survival/DFS) or death (post-metastatic survival/PMS), respectively. All deaths were tumor related.

All parameters were tested for normal distribution by the Shapiro-Wilk test. Therefore, depending on their symmetry and nature, variables were described by using mean ± standard deviation, percentage, medians with 25 and 75 percentiles or relative and absolute frequencies. The association between categorical variables was analyzed using the χ^2^ test. For statistical analysis involving quantitative variables, a non-parametric test, such as the Kruskal–Wallis’s test (the nonparametric version of the ANOVA). Bonferroni correction was performed due to multiple statistical tests. Dunn test was performed as a post hoc test after a significant Kruskal-Wallis test. Pearson’s correlation test was carried out to analyze the statistical relationship, or association, between two continuous variables. For analysis of the survival data of patients, the Kaplan-Meier curves and the log-rank test were performed. Crude and adjusted hazard ratios (HRs) and 95% confidence intervals (CIs) were calculated by using the Cox proportional hazards model. The simultaneous prognostic effect of various factors was determined in multivariate analysis by using the Cox proportional-hazard regression model. All reported *p* values are 2-sided, and values below 0.05 were considered statistically significant. All analyses were made by using IBM SPSS Statistics 25.

## 5. Conclusions

In conclusion, the data presented herein obtained from a very specific subset of patients presenting with CRC with resected CRLM revealed that altered PINK1 immunoexpression, either less than 40 points or more than 100 points by H-score determined in CRC, was a significant independent predictor of prognosis for OS and PMS. Nevertheless, these results require validation in prospective studies also, including metastatic CRCs to other locations of the body, to further support the clinical application of PINK1 immunoexpression as a useful biomarker for risk stratification. Ultimately, this data could also provide the rationale for the use of therapies to regulate PINK1 in patients with CRC and resected CRLM.

## Figures and Tables

**Table 1:** Patient demographics, histopathology, molecular features and PINK1 expression in CRCs by localization.

	Total	Right Colon	Left Colon	Rectum	*p* Value
**Patients**	90	23 (25%)	43 (48%)	24 (27%)	-
**Age (years)**	63.8 ± 9.5	64.7 ± 2.6	65.0 ± 4.8	60.8 ± 3.9	0.167
**Gender**					**0.025**
**-male**	27 (30%)	10 (43%)	7 (16%)	10 (41%)	
**-female**	63 (70%)	13 (57%)	36 (84%)	14 (59%)	
**Status**					0.437
**-alive**	46 (51%)	10 (43%)	25 (58%)	11 (46%)	
**-dead**	44 (49%)	13 (57%)	18 (42%)	13 (54%)	
**Tumor size (cm)**	4.3 ± 2.8	4.6 ± 2.4	4.6 ± 3.1	3.4 ± 1.7	**0.029**
**Nodal stage**					0.911
**-N0**	44 (49%)	12 (52%)	23 (53%)	11 (46%)	
**-N1–3**	46 (51%)	11 (48%)	20 (47%)	13 (54%)	
**Tumor stage**					0.910
**-I/II**	44 (49%)	12 (52%)	21 (49%)	11 (46%)	
**-III/IV**	46 (51%)	11 (48%)	22 (51%)	13 (54%)	
**Survival (months)**					
**-OS**	87.4 ± 9.8	72.8 ± 10.9	87.8 ± 18.4	86.2 ± 14.3	0.931
**-DFS**	39.1 ± 6.0	29.2 ± 7.7	36.3 ± 5.7	43.7 ± 11.1	0.773
**-PMS**	69.7 ± 9.0	50.7 ± 9.0	72.7 ± 15.6	71.2 ± 13.5	0.694
**Neoadjuvant therapy**					**<0.001**
**-No**	69 (77%)	22 (96%)	36 (84%)	11 (46%)	
**-Yes**	21 (23%)	1 (4%)	7 (16%)	13 (54%)	
**Tumor buds**					0.529
**-<10**	24 (27%)	7 (30%)	12 (28%)	5 (21%)	
**-≥10**	65 (73%)	16 (70%)	31 (72%)	19 (79%)	
**Grade**					0.240
**-Well**	68 (76%)	17 (74%)	35 (81%)	16 (66%)	
**-Moderate**	14 (17%)	4 (17%)	7 (17%)	4 (17%)	
**-Poor**	6 (7%)	1 (9%)	1 (2%)	4 (17%)	
**Necrosis**					**0.004**
**-No**	22 (24%)	4 (17%)	8 (19%)	10 (42%)	
**-<25%**	39 (44%)	13 (57%)	22 (51%)	4 (17%)	
**-25%/50%**	20 (22%)	6 (26%)	10 (23%)	4 (17%)	
**-50%/75%**	3 (3%)	0	0	3 (12%)	
**->75%**	6 (7%)	0	3 (7%)	3 (12%)	
**Mitotic activity (per 10 HPF)**					
**-CRC**	38.2 ± 12.8	40.0 ± 11.3	38.5 ± 8.7	35.9 ± 10.6	0.903
**-CRLM**	39.3 ± 10.6	24.8 ± 12.5	38.2 ± 8.1	43.5 ± 8.4	0.470
**MSI status**	47 (52%)	11 (48%)	27 (63%)	9 (38%)	**0.045**
**-Preserved**	47 (100%)	11 (100%)	27 (100%)	9 (100%)	
**-Altered**	0	0	0	0	
**All-Ras status**	56 (62%)	15 (65%)	29 (67%)	12 (50%)	
**-Wild type**	25 (45%)	3 (20%)	14 (48%)	8 (67%)	
**-Mutated**	31 (55%)	12 (80%)	15 (52%)	4 (33%)	
**PINK1 immunoexpression**					
**-CRC**	82.3 ± 48.7	79.0 ± 49.9	76.4 ± 39.6	95.6 ± 60.6	0.465
**-CRLM**	91.3 ± 49.6	105.0 ± 70.8	95.5± 42.1	71.3 ± 30.8	**0.018**

Values in bold are statistically significant, *p* < 0.05. OS: Overall survival; DFS: Disease-free survival; PMS: Overall survival after metastasis; HPF: High power field; CRC = colorectal adenocarcinoma; CRLM = colorectal liver metastases; MSI = Microsatellite Instability.

**Table 2:** Histopathological features and PINK1 immunoexpression of CRC and CRLM by localization.

	Right Colon	Hepatic Metastasis	*p* Value	Left Colon	Hepatic Metastasis	*p* Value	Rectum	Hepatic Metastasis	*p* Value
**Tumor buds**			0.448			**0.009**			**0.022**
**-<10**	8 (35%)	7 (30%)		12 (28%)	20 (47%)		5 (21%)	12 (50%)	
**-≥10**	15 (65%)	16 (70%)		31 (72%)	23 (53%)		19 (79%)	12 (50%)	
**Grade**			0.139			**0.027**			0.093
**-Well**	17 (74%)	15 (65%)		35 (81%)	31 (72%)		16 (66%)	16 (66%)	
**-Moderate**	5 (22%)	6 (26%)		7 (16%)	9 (21%)		4 (17%)	6 (25%)	
**-Poor**	1 (4%)	2 (9%)		1 (3%)	3 (7%)		4 (17%)	2 (9%)	
**Necrosis**			0.477			0.250			0.310
**-No**	3 (13%)	2 (9%)		7 (16%)	3 (7%)		10 (42%)	4 (17%)	
**-<25%**	14 (61%)	9 (39%)		23 (54%)	12 (28%)		4 (17%)	3 (12%)	
**-25%/50%**	6 (26%)	3 (13%)		10 (23%)	6 (14%)		4 (17%)	3 (12%)	
**-50%/75%**	0	3 (13%)		0	10 (23%)		3 (12%)	2 (9%)	
**->75%**	0	6 (26%)		3 (7%)	12 (28%)		3 (12%)	12 (50%)	
**Mitotic activity (per 10 HPF)**	40.0 ± 11.3	36.9 ± 14.5	0.709	38.5 ± 8.7	38.9 ± 9.6	0.889	35.9 ± 10.6	43.52 ± 14.7	0.200
**PINK1 expression**	79.0 ± 49.9	105.0 ± 70.8	**0.012**	76.4 ± 39.6	95.5± 42.1	**0.015**	95.6 ± 60.6	71.3 ± 30.8	0.084

Values in bold are statistically significant, *p* < 0.05. HPF: High power field.

**Table 3:** Pearson’s correlation test for clinicopathological data and PINK1 immunoexpression in CRC and CRLM.

	Age	Tumor Size	OS	DFS	PMS	Mitotic Activity CRC	Mitotic Activity in Liver Metastasis	PINK1 in CRC
**Tumor size**	r = 0.079 *p* = 0.461							
**OS**	r = −0.184 *p* = 0.82	r = −0.011 *p* = 0.922						
**DFS**	**r = −0.213** ***p* = 0.044**	r = −0.006 *p* = 0.957	**r = 0.412** ***p* < 0.001**					
**PMS**	r = −0.098 *p* = 0.360	r = −0.027 *p* = 0.798	**r = 0.863** ***p* < 0.001**	r = −0.092 *p* = 0.390				
**Mitotic activity in mCRC**	**r = 0.227** ***p* = 0.035**	r = 0.030 *p* = 0.786	r = 0.019 *p* = 0.859	r = −0.096 *p* = 0.378	r = 0.069 *p* = 0.529			
**Mitotic activity in CRLM**	r = 0.058 *p* = 0.602	r = 0.009 *p* = 0.937	r = −0.033 *p* = 0.771	r = 0.013 *p* = 0.908	r = −0.025 *p* = 0.824	r = 0.085 *p* = 0.457		
**PINK1 in CRC**	r = −0.066 *p* = 0.542	r = −0.060 *p* = 0.581	r = −0.139 *p* = 0.197	r = 0.018 *p* = 0.869	r = −0.099 *p* = 0.360	r = −0.108 *p* = 0.324	r = 0.213 *p* = 0.056	
**PINK1 in CRLM**	r = 0.106 *p* = 0.327	r = 0.148 *p* = 0.169	r = −0.101 *p* = 0.351	r = −0.001 *p* = 0.992	r = −0.118 *p* = 0.274	r = 0.124 *p* = 0.261	r = 0.050 *p* = 0.660	**r = 0.352** ***p* < 0.001**

Values in bold are statistically significant, *p* < 0.05; OS: Overall survival; DFS: Disease-free survival; PMS: Overall survival after metastasis; r = Pearson’s correlation coefficient; CRC = colorectal adenocarcinoma; CRLM = colorectal liver metastases.

**Table 4:** Univariate and multivariate model for the effect of PINK1 expression on OS and PMS in CRC with resected CRLM.

Variables	OS			PMS		
	HR	95% CI	*p* Value	HR	95% CI	*p* Value
**PINK1 in CRC,** **score (<40 or >100 vs. 40–100)** **-Univariate** **-Multivariate**	2.090 1.972	1.035–4.219 0.971–4.005	**0.015** **0.022**	2.055 2.023	1.014–4.164 1.003–4.091	**0.026** **0.037**
**Age, years** **(≤60 vs. >60)**	0.541	0.273–1.072	0.078	0.642	0.324–1.276	0.204
**Gender** **(male vs. female)**	0.769	0.399–1.483	0.433	0.763	0.398–1.464	0.416
**Neodjuvant therapy** **(Yes vs. no)**	2.111	1.030–4.326	**0.041**	0.635	0.304–1.079	0.203
**Stage at diagnosis** **(I–II vs. III–IV)**	0.900	0.493–1.642	0.731	1.290	0.703–2.368	0.410
**Grade in CRC** **(Poor vs. well to moderate)**	1.620	1.067–2.459	**0.024**	0.572	0.304–1.079	0.084
**Tumor size in CRC** **(≤4.3 cm vs. >4.3 cm)**	0.780	0.384–1.585	0.492	0.892	0.384–1.585	0.712
**Tumor buds in CRC** **(<10 vs. ≥10)**	0.343	0.144–0.818	**0.016**	0.364	0.153–0.863	**0.022**
**All-Ras status** **(Wild type vs. mutated)**	1.136	0.532–2.432	0.742	0.995	0.464–2.134	0.991

Cox regression model. OS: Overall survival; PMS: Overall survival after metastasis; CI: confidence interval; HR: hazard ratio: CRC: colorectal adenocarcinoma. Values in bold are statistically significant, *p* < 0.05.

## Data Availability

The data that support the findings of this study are available on request from the corresponding author. The data are not publicly available due to privacy or ethical restrictions.
